# Analysis of ^18^O/^16^O Isotope Ratios in Organic Matter by Laser Ablation IRMS

**DOI:** 10.1021/acs.analchem.4c06896

**Published:** 2025-03-27

**Authors:** Elina K. Sahlstedt, Neil J. Loader, Katja T. Rinne-Garmston

**Affiliations:** † Natural Resources Institute Finland (Luke), Latokartanonkaari 9, 00790 Helsinki, Finland; ‡ Department of Geography, Swansea University, Singleton Park, Swansea SA2 8PP, Wales, U.K.

## Abstract

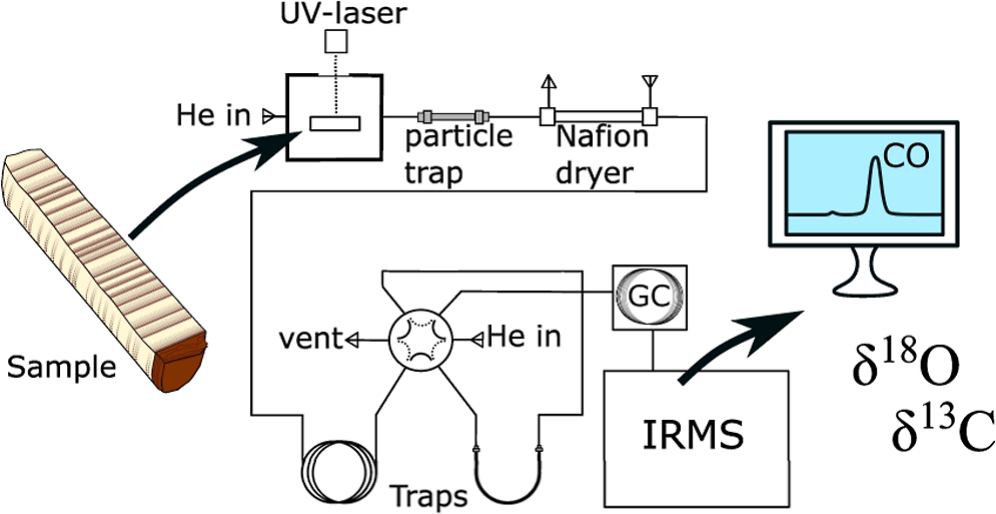

In recent years, the application of laser ablation isotope ratio mass spectrometry has revolutionized the field of carbon isotope analysis, enabling routine analyses at high spatial resolution (30–40 μm). Until now, an equivalent analytical method for oxygen isotope ratio (^18^O/^16^O) measurements has been lacking. In this article, we describe a preparatory system for analysis of the oxygen isotope composition of carbon monoxide produced from organic samples. The system couples a UV laser platform and automated cryofocusing unit with an isotope ratio mass spectrometer. The method is tested on cellulose and wood (tree rings) and is shown to produce data with analytical precision typically better than 0.4‰ with a sampling resolution of 100 μm (laser beam diameter). Coupled with spatially accurate and minimally invasive laser sampling, the ability to measure stable oxygen isotopes in this way represents a significant advance as it opens up new research opportunities in plant sciences, ecology, paleoclimatology, and science-based archeology.

## Introduction

The stable oxygen isotope composition of organic materials such as wood, calcite, bone, and tooth provides an important source of environmental information and a powerful natural tracer for investigating physiological processes, ecosystem fluxes, and environmental change. In natural archives that form annual increments including tree rings and the growth bands in shells, corals, speleothems, and otoliths, it is possible to sample within each annual increment, to develop seasonally resolved records of isotopic variability, and to link these records to environmental and physiological controls.^1−5^

Measuring isotope compositions within an annual increment is an exacting task requiring the sequential subsectioning of relatively large samples. While manual division is possible, subsampling is typically conducted using a microtome,^6^ micromill,^7^ or laser microdissector.^8^ Each subsample must then be prepared for mass spectrometry. This may include additional chemical pretreatments, followed by the weighing and packing of each subsample into tin/silver capsules for combustion/pyrolysis. These techniques have led to significant advances in understanding, but they are resource intensive and demand a high level of operator expertise. This limits the application of the method to samples with large increments and typically low levels of sample replication result. To fully exploit the research potential of intra-annual archives, there is a need to develop more rapid and cost-effective approaches that will enable the development of well-replicated, high-resolution data sets.

In recent years, the application of laser ablation isotope ratio mass spectrometry (LA-IRMS) has transformed the field of intra-annual carbon isotope analysis.^9−12^ In this method, a sample (most often a tree core) is collected by laser ablation, and the ablated material is then transferred from the sample chamber to a miniaturized combustion unit on a flow of helium gas. Here, it is converted to carbon dioxide (CO_2_) for analysis by IRMS.^13−17^ This laser ablation approach has enabled high spatial resolution (30–40 μm) intra-annual analysis of the C-isotope composition of tree rings.^14^ Compared with conventional analysis methods, the laser ablation approach offers very high-resolution analyses, with rapid sample throughput, improved versatility, and simplified sample preparation.

Until now, however, a similar, high-resolution analytical system for oxygen isotope (^18^O/^16^O) ratio measurements in tree rings has been lacking. This partly reflects challenges that are similar to those encountered during the development of online oxygen isotope analysis,^18^ but there are additional considerations relating to the chemical composition of wood, sample transfer, and fractionation that have further hampered progress.

A recent study of laser ablation of wood and cellulose for carbon isotope analysis^14^ and organic materials more generally^19−23^ has suggested that in addition to particulate matter, the laser ablation process forms gaseous phases, with only a minority of it being CO_2_.^14^ The relative proportion of gas to particles produced is directly linked to the O/C ratio of the ablated substance, where a higher sample O/C ratio, e.g., ∼0.7 for wood and cellulose, tends to induce more gas formation.^19^ In the oxygen-free atmosphere of the laser sample cell, CO is therefore the likely primary product when samples contain carbon and oxygen. If this conversion to CO can be shown to be reproducible, then it would be possible to produce samples of CO for oxygen isotope analysis directly via laser ablation without the need for manual dissection or off-line preparation.

In this paper, we describe a method of measuring the ^18^O/^16^O ratios of organic samples using a UV laser ablation device coupled with an automated cryo-focusing unit and isotope ratio mass spectrometer. We also evaluate the possibility of dual measurement of the ^18^O/^16^O and ^13^C/^12^C ratios using the new method.

## Methods

### Overview of Instrumentation

**Figure 1:**
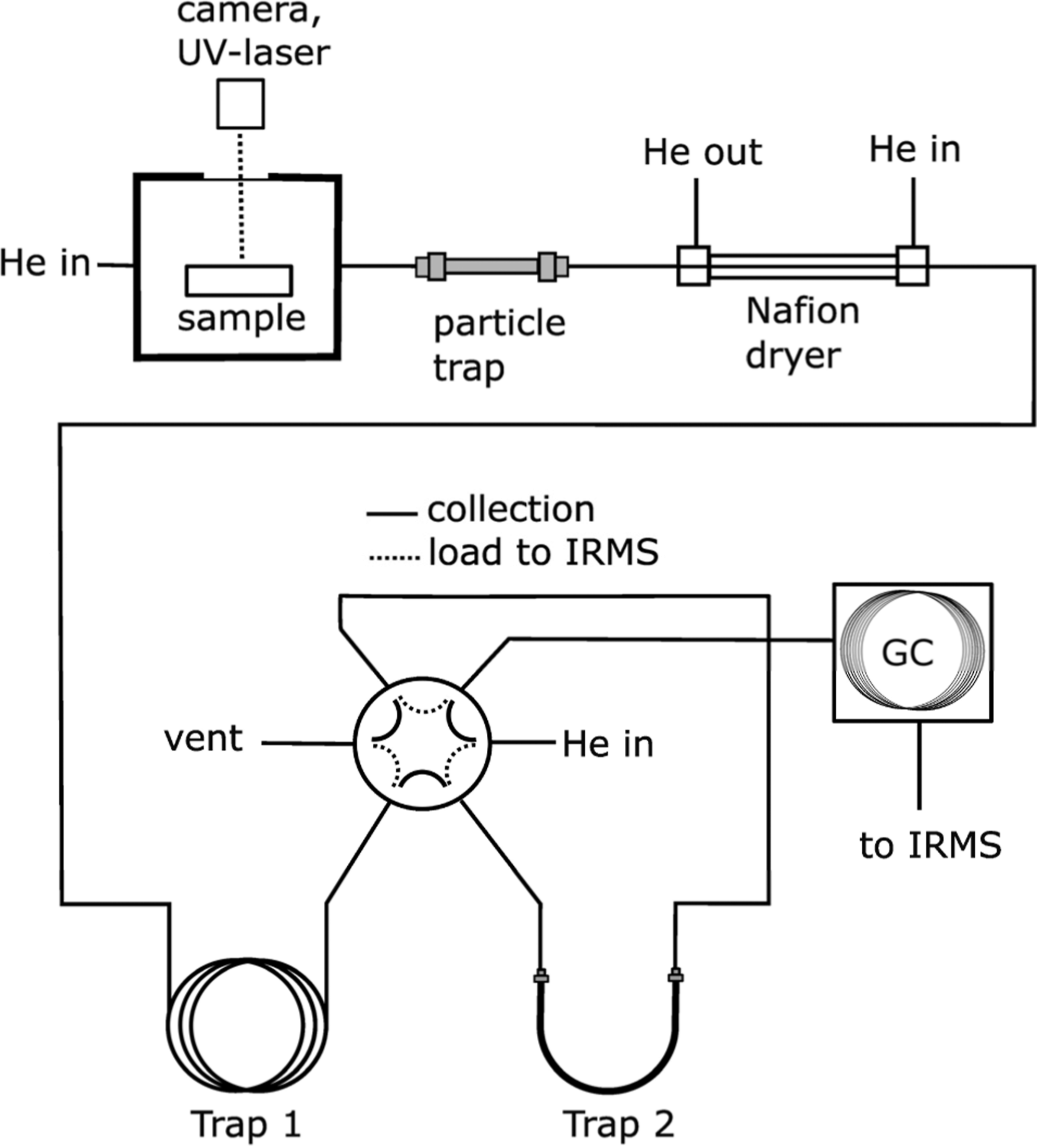
Schematic outline of the instrument used for the O-isotope analyses. The particle trap consists of a 10 cm 1/8″ stainless steel tubing into which quartz wool has been inserted. Water is removed from the sample stream by a Nafion dryer. Trap 1 collects condensable gases such as CO_2_, while Trap 2 collects CO gas from the sample and N_2_ gas originating from the background. Gases in Trap 1 are vented into the atmosphere via a 6-way valve, while gases in Trap 2 are first loaded into the GC-separation column (5 Å Molecular sieve PLOT column) where N_2_ is separated from CO prior to the arrival at the IRMS.

The main features of the instrumentation are as follows: a laser ablation unit (213 nm Nd:YAG laser, Teledyne Photon Machines) with a laser ablation cell (IsoSCell, TerraAnalitic), connected via a gas trapping and concentration unit (Cryoprep, Sercon Ltd.) to an IRMS (model: 20–22, Sercon Ltd.). The laser ablation unit is operated via a computer, with a second computer controlling the operation of the Cryoprep and the IRMS. This basic configuration is similar to those previously used for carbon isotope analysis.^14^ The following modifications were implemented as follows to enable the analysis of stable oxygen isotopes (Figure 1).

#### Particle Trap

A particle trap was installed downstream of the laser ablation cell to prevent any particulate matter produced by or released during the ablation process from entering the Cryoprep unit or mass spectrometer. This physical trap consists of an approximately 10 cm long 1/8 in. diameter stainless steel tube (LUKE) or 2 cm 1/8 in. diameter silica tube (Swansea) with fine quartz wool inserted into it. It is connected to the outlet of the laser chamber by a length of 1/16 in. PEEK tubing, and to the inlet of the Cryoprep unit by a length of 1/16 in. stainless-steel tubing. The particle trap needs to be periodically cleaned; specifically replacing the quartz wool and removal of any adhered dust particles using compressed air. In this method, the laser produces the CO that is analyzed, but the particulate filter is still required as we have observed the release of physical particles (sawdust) and cellulose fibers from the sample and reference during the ablation process which could restrict gas flows and damage the analytical system.

#### Water Removal

After removal of particulates, the sample gases are carried on a flow of helium (adjustable using the laser controlling software) into the Cryoprep unit. The gas stream flows first through a Nafion gas dryer (Perma Pure), which removes H_2_O from the sample stream.

#### Cryotrapping Condensable Gases

The first cryotrap (Trap 1) consists of four loops (approximately 150 cm total length) of 1/16 in. stainless steel tubing. It is used to trap condensable gases, such as CO_2_, which are formed in minor amounts during laser ablation.^14^ The condensable gases are released later during the analytical run and vented to the atmosphere via a 6-way valve (Figure 1).

#### CO Trapping

As the freezing point of the CO gas is below that of liquid nitrogen, the CO produced by the laser passes freely through the first cryotrap. The CO then enters the second trap (Trap 2) via the 6-way valve. The second trap is modified to cryogenically trap and focus the CO gas and comprises a U-shaped section of 1/8 in. stainless-steel tubing (approximately 30 cm length). At the bottom of the loop is a filling of 5 Å molecular sieves (80–100 mesh, Elemental Microanalysis) held into place on either side by a small amount of quartz wool. We used 1.5 cm of filling (approximately 0.1 mL) at the bottom of the loop. Two pieces of 1/16 in. peek tubing were used to insert and correctly position the quartz wool plugs at the bottom of the loop and to gently pack the molecular sieve grains into place. The CO trap is connected to the 1/16 in. stainless-steel tubing by 1/16 in. to 1/8 in. stainless-steel reducing union (Swagelok). Both trapping loops are lowered and lifted from a Dewar containing liquid N_2_ by pneumatically operated solenoids.

#### Separation of CO from N_2_

Following capture of the CO, Trap 2 is removed from the liquid N_2_ and allowed to thaw. The gas flow is then redirected via the 6-way valve into a CG-column suitable for separating CO from potentially interfering (background) gases, especially N_2_. This is achieved using a 5 Å Molecular sieve PLOT column (15 m, I.D. 0.53 mm, df 50 μm, Restek GC columns). The helium flow and GC column temperature can be adjusted to optimize the separation of CO (Figure S1, Supporting Information), as described below.

### Analysis Sequence for O Isotope Measurement

The analysis sequence for the ^18^O/^16^O ratio measurement lasts for 900 s (LUKE) and 720 s (Swansea), adjusted according to the arrival of the sample signal in the chromatogram, and is controlled by the Callisto program of the IRMS instrument (version 3110, Sercon Ltd.). At the start of the sequence (5 s), Traps 1 and 2 are lowered into the liquid N_2_-filled Dewar. The helium flow through the laser chamber and into the traps, controlled by the laser operating software, is set to 30 mL min^–1^. Once the traps have cooled (5 s), Callisto triggers the laser operating program (Chromium, version 2.4, Teledyne Photon Machines) to start the ablation process. We used laser power at 45–60% and a spot size of 100 μm, run along a line of 0.6–1.0 mm. This configuration was tested to provide sufficient signal size for precise ^18^O/^16^O ratio measurement. After a predetermined collection period of 100 s, the 6-port valve switches to the alternative configuration and the two traps are removed from the liquid nitrogen and allowed to thaw (Trap 1 at 102 s, Trap 2 at 110 s). The initial 30 mL min^–1^ carrier flow is then directed through Trap 1 to atmosphere, and the contents of Trap 2 are released into the GC column. The helium flow through Trap 2 (controllable via a needle valve) is set to approximately 3 mL min^–1^ at this stage.

In addition to trapping CO produced by the laser, our tests have shown that traces of N_2_ are also trapped on the 5 Å molecular sieve. We suggest that this N_2_ (or oxides of nitrogen) originates primarily through the ingress of atmospheric nitrogen via the sample cell with a small contribution from nitrogen contained within the sample. It is important that any trapped N_2_ is separated from the CO prior to analysis. As the traps warm to room temperature, condensable gases (Trap 1) vent into the atmosphere, while CO and N_2_ (Trap 2) are separated as they pass through the GC column, which is maintained at an optimal temperature of 30–35 °C (Figures 1 and S1). If desired, the mass spectrometer may be isolated from the Cryoprep unit until the N_2_ gas has eluted from the GC column to ensure that no N_2_ enters the ion source.

The mass signals 28, 29, and 30 of the CO are monitored by the IRMS operating software Callisto. The software calculates the ^18^O/^16^O ratios of the samples based on the integrated peak areas and returns the oxygen isotope composition of the samples as δ^18^O values, given in per mille, defined as
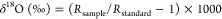
where *R* is the ^18^O/^16^O ratio. In the Callisto program, each analytical run conducted in the “normal” mode includes one or more analyses of a single reference material designated as “references”. Based on these reference points, the program calculates the δ^18^O values for subsequent measurements. The program also automatically calculates and corrects for instrumental drift based on the set “reference” measurement points.

In addition to δ^18^O values, Callisto software also calculates the ^13^C/^12^C ratios from the CO mass signals in a similar manner as the δ^18^O values, based on the known δ^13^C values of the reference materials. This allowed us to monitor the δ^13^C data produced by the new method and estimate whether the new method could also be utilized for the dual analysis of the δ^18^O and δ^13^C values.

**Figure 2:**
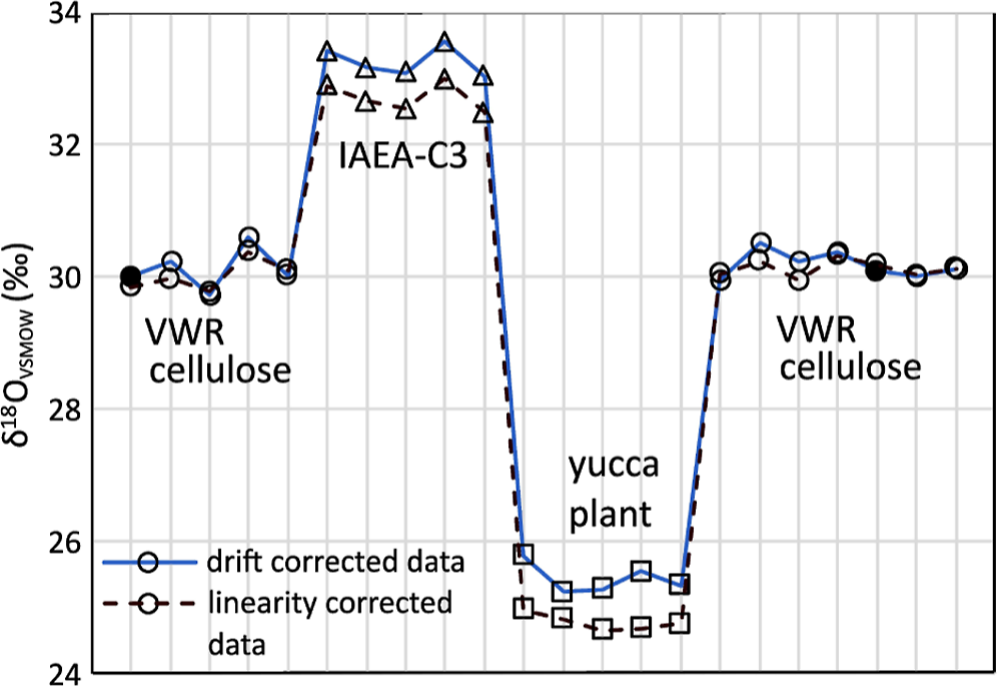
Example run sequence (*x*-axis, data from a single continuous sample run) for materials with distinct δ^18^O values: VWR cellulose (expected δ^18^O = 30.0‰, measured, linearity corrected δ^18^O = 30.2 ± 0.3‰), IAEA-C3 (expected δ^18^O = 32.6‰, measured, linearity corrected δ^18^O = 32.7 ± 0.2‰), and yucca plant (expected δ^18^O = 24.6‰, measured, linearity corrected δ^18^O = 24.8 ± 0.1‰), to demonstrate the absence of a memory effect between samples. The solid circles indicate the VWR cellulose reference points, which the Callisto program (Sercon Ltd.) uses to calculate (raw) δ^18^O values and perform drift correction. Additionally, the figure includes these data with a linearity correction for comparative purposes (dashed line).

The performance of the system was tested using several materials including IAEA-C3 cellulose and VWR cellulose pads (Blotting pads, Cat. number 732-0601), USGS-55 Mexican ziricote wood powder (δ^18^O = 19.1 ± 0.1‰), and an internal standard made from yucca plant powder, which was less homogeneous than the cellulose samples. Prior to laser sampling, the wood powders were compressed into solid tablets (13 mm diameter) by using a manual hydraulic press (Table 1 and Figure 2). The IAEA-C3 cellulose used in this study has an expected δ^18^O value of 32.6‰.^24^ VWR cellulose and yucca plant material have previously been analyzed in our laboratory using thermal conversion IRMS, calibrated against IAEA-601 and two in-house reference materials (lactose = 21.1 ± 0.2‰, sucrose = 36.6 ± 0.2‰). Based on these data, the expected values for these working standards are 30.0 ± 0.2‰ (*n* = 10) for VWR cellulose and 24.6 ± 0.2‰ (*n* = 12) for yucca.

**Table 1:** Results for Analyses of Reference Materials, Covering Several Separate Runs

ID	δ^18^O_raw_ (‰)^a^	δ^18^O_lin_ (‰)^b^	δ^18^*O*_exp._ (‰)^c^
IAEA-C3	33.1 ± 0.4 (*n* = 21)	32.8 ± 0.3 (*n* = 21)	32.6^d^
USGS-55	21.3 ± 1.0 (*n* = 18)	19.5 ± 0.3 (*n* = 18)	19.1 ± 0.1
VWR	29.9 ± 0.9 (*n* = 30)	30.1 ± 0.3 (*n* = 30)	30.0 ± 0.2 (*n* = 10)
yucca	24.7 ± 0.6 (*n* = 25)	24.5 ± 0.4 (*n* = 25)	24.6 ± 0.2 (*n* = 12)

a Raw values are drift corrected by the Callisto program.

b Linearity-corrected data.

c Expected values.

d The value is from Boettger et al.^24^

To evaluate our method further, tests were conducted on a lath of resin extracted from wood from a pine tree (*Pinus sylvestris* L.) located in Hyytiälä, southern Finland. This sample had been previously analyzed using conventional methods to determine its δ^18^O values at intra-annual resolution.^25^ We also analyzed an oak sample (*Quercus* spp) from Oxfordshire in the southern United Kingdom at an annual resolution. As oak is a nonresinous species, tests were conducted on the whole wood without resin extraction. In both cases, an even surface, suitable for laser focusing, was prepared by sanding. Prior to analysis, areas for analysis were preablated with a low energy laser sweep to help remove any possible contaminants and particulates (e.g., wood powder) that may have adhered to the sample surface.

## Results and Discussion

In this section, we present and discuss the performance of the new method as applied to reference materials and two case studies. We also explore the potential for simultaneously analyzing carbon isotope composition from the laser-produced CO, and we identify areas for future development and optimization of the method.

### Precision and Accuracy

The precision and accuracy of the method was tested by running series of analyses alternating between different standard materials. In a typical run, the precision of measurement for samples of similar size was 0.3–0.4‰ (σ_*n*–1_*n* = 5). In the current experimental setup, we observed a size effect (or linearity effect) in the data (Figure 2 and Table 1). This results from sample matrix effects (differences in density/energy dispersal) and the chemical composition of the test materials which can influence the amount of CO produced during ablation. Two simple strategies may be applied to counter this: (1) adjusting the ablation track lengths (or laser spot size) to ensure similar amounts of CO are produced from different sample materials and (2) collecting a set of data with different signal sizes (by adjusting ablation track lengths) which is then used to correct the data for sample size effects. Using these strategies improved the precision of the measured ^18^O/^16^O ratios with the δ^18^O values across all reference materials falling within 0.4‰ of their reported values (Table 1). Further, we tested the background/blank of the analyses by running sample sequences without ablation, as this would show the presence of a potential interfering signal arriving at the same time as the sample CO peak (Supporting Information). No measurable peaks were detected.

### Analysis of Hyytiälä Pine

**Figure 3:**
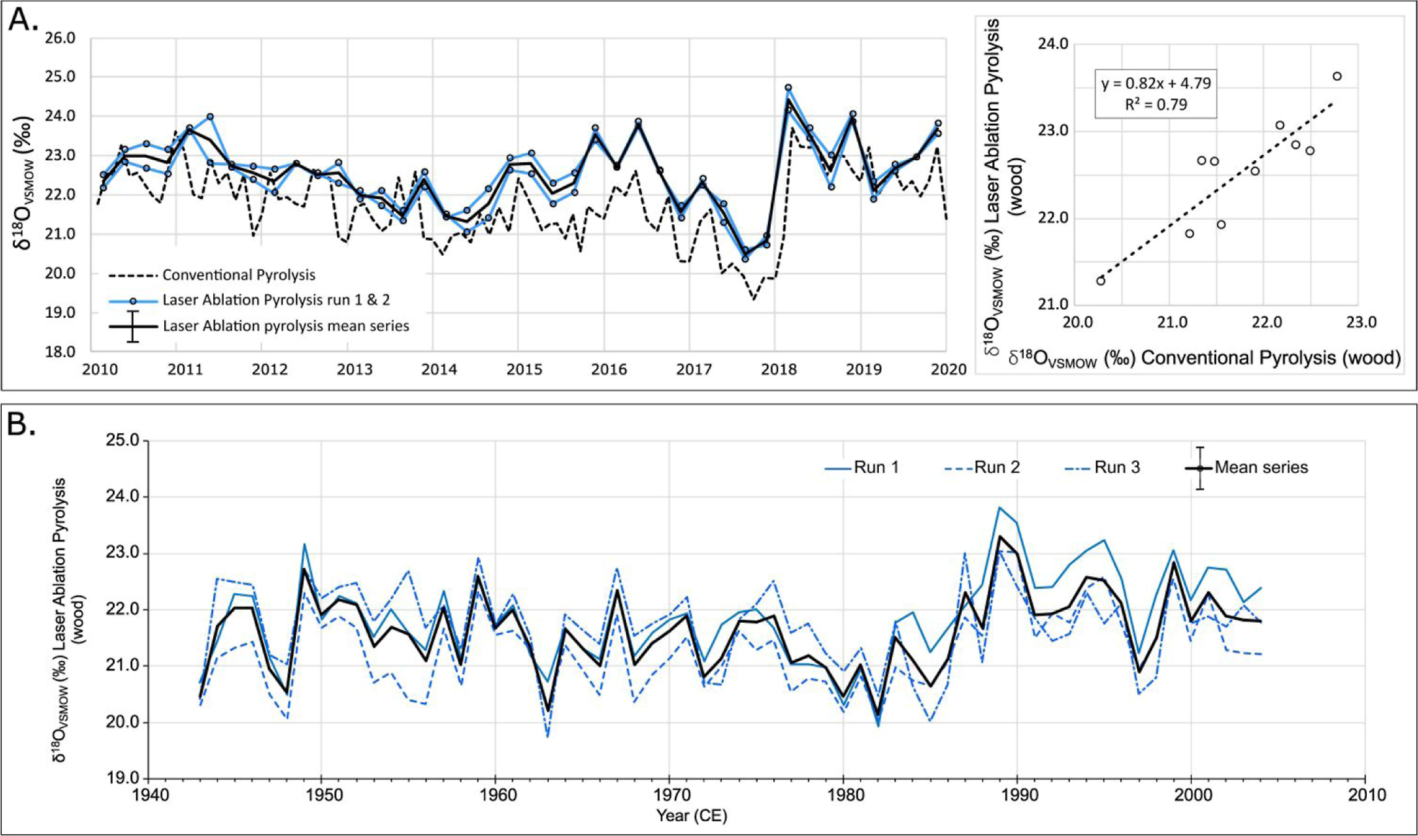
(A) Hyytiälä pine. The graph compares intra-annual wood sections analyzed by thermal conversion IRMS (Conventional Pyrolysis, dashed line, data from ref (25)) with data acquired using the new method (two runs, represented by blue lines, laser ablation pyrolysis). Data corrected for drift (by Callisto) and linearity (post processing in Excel) and normalized to IAEA-C3 (set value 32.6‰). Estimated measurement error of laser ablation pyrolysis measurements is shown on the legend (±0.4‰, typical standard deviation of measurement of reference materials and wood). Surface of the analysis lines were preablated with a low-energy (3%) laser. Right panel shows the comparison between the conventional and new data, when reduced to annual increments. NB: Sampling resolution differs between the thermal conversion and laser ablation analyses. (B) English oak. δ^18^O values obtained from oak late wood by the new Laser Ablation Pyrolysis method (whole wood). A 62 year long series was measured, each year in triplicate (individual runs in blue, mean series in black). Error bar shown on the legend (Mean series) indicates mean standard deviation of measurements (0.4‰, min 0.1‰, max 1.1‰).

We determined the intra-annual variation in δ^18^O in samples of Scots Pine (*P. sylvestris*) from the Hyytiälä Research Station, Finland, using the new laser ablation method and compared the results with those obtained from conventional methods, which involved analyzing microdissected wood laths^25^ (Figure 3A). Both sets of analyses were conducted on resin-extracted wood without any additional chemical sample preparation. The laser ablation analyses were conducted on a different core from the same tree and run twice over the years 2010–2019. The direction of the analyses is the same, i.e., perpendicular to the growing direction of the ring, but the sampling resolutions are different (higher for conventional analyses, where samples were cut with a cryo-microtome^25^). The new method successfully captures similar intra-annual variation in δ^18^O values, particularly the increase in δ^18^O from year 2017 to 2018 (*r* = 0.88, *n* = 40). We observe an offset in the δ^18^O values between the two studies, with a mean offset of 0.8 ± 0.3‰, calculated for annually resolved data. This likely reflects real isotopic differences around the circumference of the sampled tree,^26^ given that the laser ablation and microdissection analyses are performed on different cores. The data compare favorably when the sampling resolution is reduced to annual increments (*r* = 0.89, *n* = 10) (Figures 3 and S2).

### Analysis of English Oak

A sample of oak (*Quercus* spp.) sourced from a recently felled tree was analyzed using the laser ablation system. Oak is a ring porous species, so sampling was conducted on the latewood fraction only. Measurements were performed in triplicate on a 62 year sequence of covering the years 1943–2004 CE. The three measurement series compare favorably with a mean interseries correlation of 0.72 and a mean standard deviation for the 62 triplicate measurements of 0.4 per mille (Figure 3B).

### Carbon Isotope Composition

In a growing number of isotope laboratories, the carbon and oxygen isotope composition of CO is measured simultaneously on the same analyte.^27−29^ This dual measurement of both isotopes significantly reduces analysis time and, depending on the O/C ratio of the sample material, can provide a series close to that obtained through the combustion of the sample to CO_2_. One limitation of measuring carbon isotopes (^13^C/^12^C ratios) by pyrolysis is that some carbon from the sample remained in the reactor. Subsequent samples may potentially react with this residual sample carbon and/or the glassy carbon reactor.^28^ This requires careful monitoring and may need correction for the measured ^13^C/^12^C ratios.^28,29^ By contrast, in laser ablation, carbon in the CO is derived directly from the sample material. Analogous to the oxygen isotope measurements, the CO produced during laser ablation could also be used to measure the carbon isotope composition of the sample.

To test this, we monitored the carbon isotope data produced by the instrument. Similar to the δ^18^O data, we observed no memory effects in the δ^13^C values of reference materials with distinct carbon isotope compositions (Figure S3). In contrast to the δ^18^O data, the ^13^C/^12^C measurements were not strongly affected by linearity issues.

**Figure 4:**
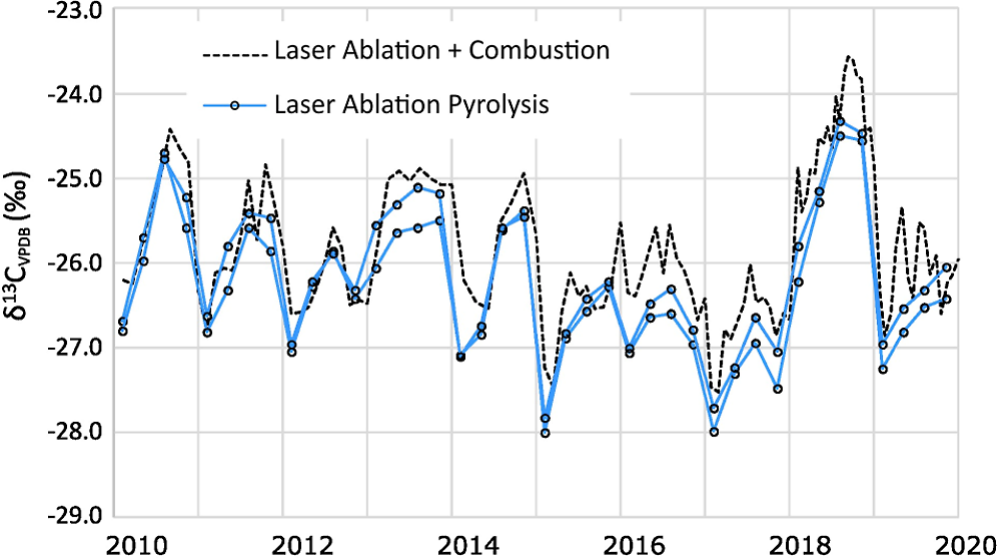
Intra-annual δ^13^C data from Hyytiälä pine obtained with the new (oxygen) method (dashed lines, laser ablation pyrolysis) compared to data from Tang et al.^9^ (solid line. Laser ablation + combustion). Data from Tang et al.^9^ was obtained with the LA-IRMS method for C-isotope analysis, which includes a combustion step. The figure demonstrates ability of the new method to reproduce intra-annual δ^13^C variation. Correlation between the δ^13^C values of the different methods when reduced to the annual scale is r = 0.97 (*n* = 10).

Figure 4 compares the intra-annual δ^13^C values of Hyytiälä pine tree obtained with the new method presented here to those acquired by the original LA-IRMS method designed for carbon isotope analysis.^9^ Overall, the new method reproduces the tree core’s intra-annual δ^13^C variation. It should be noted that the original LA-IRMS (carbon) method reports δ^13^C data measured at a spatial resolution higher than the δ^13^C values from the new (oxygen) method (Figure 4). The differences observed, which range from 0.1 to 0.6‰, are small when comparing the calculated annual average δ^13^C values (Table S3). As with the oxygen isotope comparison, some level of difference should be expected between these data sets as they represent data from different samples.

The δ^13^C values (Figures 4 and S4), obtained simultaneously with δ^18^O values using the new method, show promise for future development of simultaneous determination of oxygen and carbon isotopes from the laser-produced CO. However, in contrast to the carbon isotope analysis method for LA-IRMS, which quantitatively converts sample carbon from produced particles and gases to CO_2_ using a combustion unit,^14^ the new method converts sample material to CO with the aid of the laser. The observed variations in CO signal sizes between materials suggest potential matrix effects, which may also lead to matrix-related fractionation for carbon isotopes. Therefore, further investigation into the performance of the new method for carbon isotope analysis and potential matrix-related fractionation effects is necessary.

## Conclusions

In developing the LA-IRMS method for the analysis of carbon isotope composition of tree rings, Wieser and Brand^17^ anticipated that such instrumentation could also be used to allow the measurement of ^18^O/^16^O ratios. In our method, the laser-produced CO is cryogenically trapped with a 5 Å molecular sieve and analyzed for δ^18^O with an analytical precision of at least 0.4‰ (100 μm beam diameter) and without the need for a separate secondary pyrolysis. This level of performance is similar to current online methods and provides secure proof of concept for the future development and refinement of the technique.

Laser sampling is minimally invasive compared to conventional IRMS methods (our tests required 100 μm wide and ∼1 mm long ablation tracks), meaning that it is now possible to analyze small samples, such as microcores and very narrow ringed samples. This is particularly advantageous for unique samples or those from museum collections and ongoing experimental settings where repeated sampling may not be desirable or practical. The method also holds potential applications in paleoclimatology and science-based dating, providing rapid analyses and prescreening before undertaking more resource-intensive cellulose preparation.

Analysis time per sample run is approximately 15 min, which, although longer than standard online methods (8 min), represents a significant advance, particularly for intra-annual analyses, considering the time required for sample preparation.

The production of CO during ablation offers the possibility for measuring carbon and oxygen isotopes simultaneously, contingent on the sample’s chemistry (C/O ratio and composition). Our initial results are encouraging, highlighting this as a promising area for further research. Given the interaction between carbon and oxygen isotopes in tracing plant-water–carbon dynamics, the benefits of the dual isotope method for plant physiology could be substantial.

There is a need to further explore matrix effects on the CO production and its potential influence on δ^18^O and δ^13^C values, as well as the requirement for standardized, laser-compatible standards to foster knowledge exchange and facilitate interlaboratory comparisons. In terms of practical refinement, while cores are currently required to be split into 40 mm long sections to fit the sample chamber, the chamber could easily be adapted to accommodate larger samples. This adjustment is feasible because the method utilizes laser-produced CO gas rather than particulates, significantly reducing concerns about sample transfer.

Oxygen isotope analyses can now be performed on organic materials rapidly and with high spatial and temporal resolution using laser-ablation IRMS. We anticipate that this minimally invasive method will open up the possibilities for oxygen isotopic analysis of new archives and sample types previously inaccessible using standard methods.

## Supporting Information Available

Further experimental observations on background measurements, matrix effects, example chromatogram, and additional data on comparison of laser ablation and microdissection methods and on simultaneous determination of carbon and oxygen isotopes (PDF)
