# Supplementary material for: Analysis of 18O/16O Isotope Ratios in Organic Matter by Laser Ablation IRMS
Source: Anal Chem. 2025 Mar 27;97(13):7271–7. doi: 10.1021/acs.analchem.4c06896 (PMC11983368; doi:10.1021/acs.analchem.4c06896)
Supplement: Supplementary file 1 — ac4c06896_si_001.pdf [file ac4c06896_si_001.pdf]

Supporting Information

Analysis of $^{18}O/^{16}O$ isotope ratios in organic matter by laser ablation IRMS

Elina K. Sahlstedt[1], Neil J. Loader[2] and Katja T. Rinne-Garmston[1]

[1] Natural Resources Institute Finland, Latokartanonkaari 9, 00790 Helsinki, Finland

[2] Department of Geography, Swansea University, Singleton Park, Swansea, SA2 8PP, Wales, UK.

Contents

Further experimental observations S1

Background measurements S2

Matrix effect S2

Comparison of laser ablation and micro-dissection methods S3

Simultaneous determination of carbon and oxygen isotopes S3

References S4

Further experimental observations

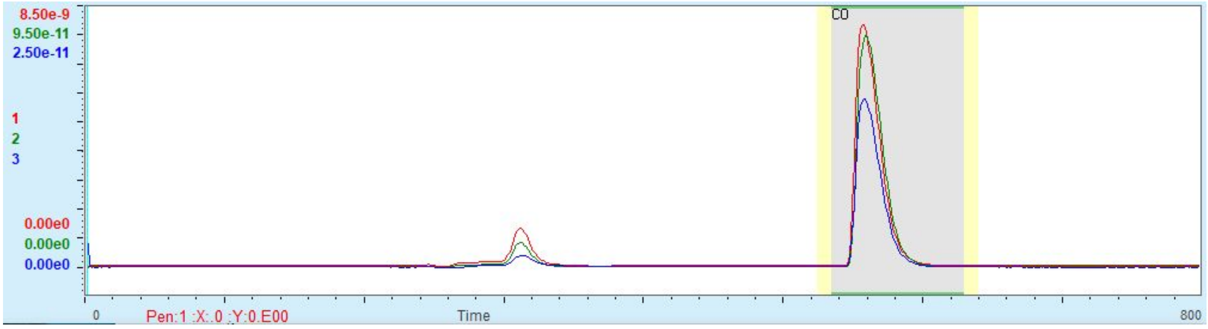

Figure S1: Example chromatograph of an analysis run. The first peak represents N_2 gas retained in the molecular sieve cryotrap (trap 2), while the second, larger peak is sample CO gas. The y-axis represents the signal intensities (A) and the x-axis the time (s).

Background measurements

We tested for an instrumental background (blank effect) by conducting analysis runs without the laser ablation step. Occasionally, but not all the time, a very small increase in background levels could be observed at the position in the chromatograph where the sample CO peak would typically be seen. As this represents <0.05% of the area of a normal sample peak, its influence is considered negligible.

Prior to the elution of the CO peak, we observe a gas peak which can be identified as N_2 based on the mass 28, 29, 30 signal ratios. The size of the N_2 peak (**Figure S1**) along with its occurrence in blank measurements (i.e., runs with no ablation), indicates that the N_2 originates from the background gas flow. This is most likely due to minor leak(s) in capillary connections and/or laser chamber connections accentuated through cryotrapping, although there may also be a contribution from nitrogen in the ablated sample. The GC-column is however very effective at separating the N_2 and the CO peaks, thereby minimizing the effects of any N_2 on CO measurements. This is supported by the analysis sequence of isotopically distinct materials that indicates no influence from memory effects (**Figure 2**).

Matrix effect

In addition to the effect of sample size, we also noted a "matrix effect" related to the ablation process, influencing the amounts of CO produced by the analyzed materials. When the ablation setting (laser energy and ablation mode, spot size and track length) were kept the same, there were differences in the CO peak sizes (amount of gas produced) depending on the materials that were being ablated. Part of this variation was likely due to differences in the density and composition of the materials. For example, wood is less dense compared to compressed powder pellets and, wood density itself varies naturally (Saurer et al., 2023). We also believe some of this variability to relate to differences in energy dispersion and C/O ratio between test samples. In day-to-day running of the system this matrix effect may be countered by adapting the laser parameters (energy, beam area, path and frequency) to match those of the other sample material.

Comparison of laser ablation and micro-dissection methods

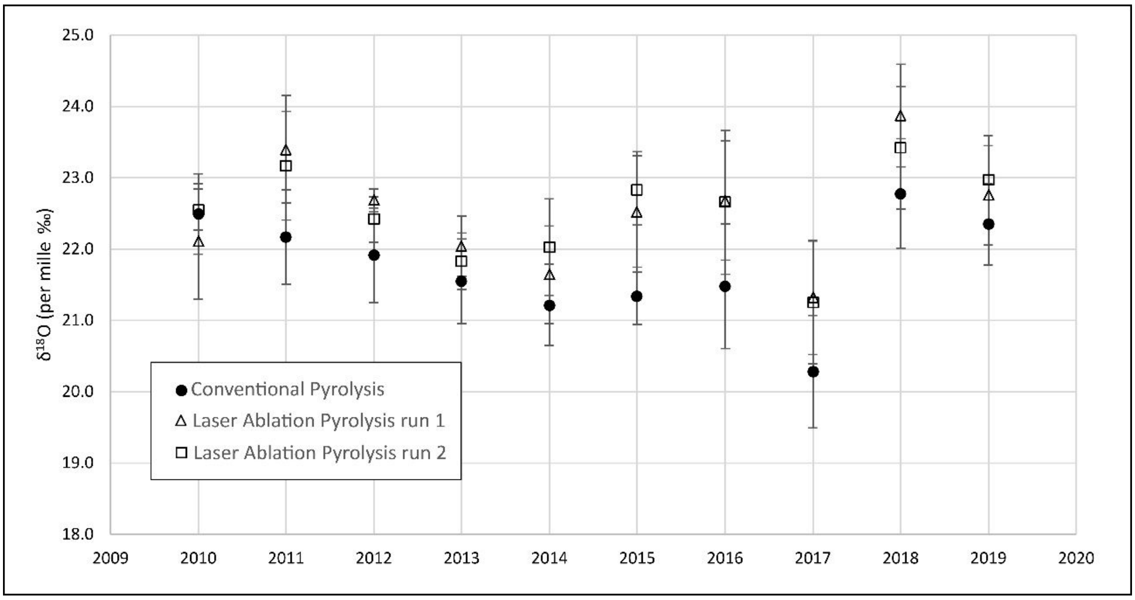

Figure S2. Annual average data for 2010-2019 obtained by thermal conversion IRMS (Conventional Pyrolysis, solid circles) and LA-IRMS method (Laser Ablation Pyrolysis, two runs, open markers). The error bars indicate standard deviation of measurements (n=8-11 for conventional, n=4 for new method).

Simultaneous determination of carbon and oxygen isotopes

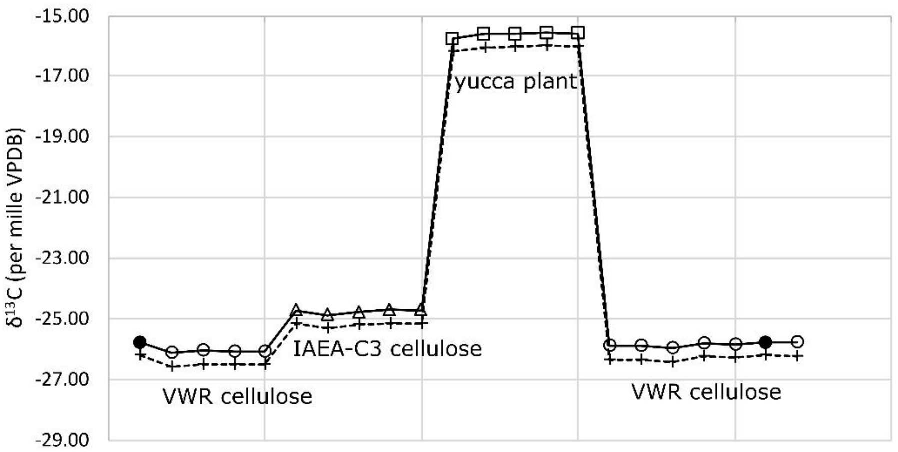

Figure S3. Carbon isotope data obtained with the method, showing a sequence of analyses of materials with distinct C-isotope composition (circles, VWR cellulose, expected $\delta^{13}C$=-26.2±0.2‰, triangles, IAEA-C3 cellulose paper, expected $\delta^{13}C$=-24.75±0.17‰, n=233, squares, yucca plant material, expected $\delta^{13}C$=-15.5±0.2‰). The raw data (crosses) was corrected for instrumental drift (automatically, by Callisto software) and normalized against the IAEA-C3 paper (measured for VWR: $\delta^{13}C$ = -26.0 ± 0.1‰, measured for yucca: $\delta^{13}C$ = -15.6 ± 0.1‰). We observe no memory effects for C isotope data.

Table S1. Average annual δ^{13}C values for years 2010-2019 for Hyytiälä tree core, obtained by Laser Ablation + Combustion method and the new method (Laser Ablation Pyrolysis) utilizing laser produced CO (data from two separate runs).

year	δ^{13}C (‰, Laser Ablation + Combustion)	stdev	δ^{13}C (‰, Laser Ablation Pyrolysis)	stdev	|difference|
2010	-25.4	0.7	-25.7	0.8	0.3
2011	-25.7	0.6	-26.0	0.5	0.2
2012	-26.3	0.4	-26.4	0.4	0.1
2013	-25.1	0.3	-25.5	0.3	0.4
2014	-25.8	0.6	-26.2	0.8	0.4
2015	-26.5	0.6	-26.9	0.7	0.4
2016	-26.1	0.3	-26.7	0.3	0.6
2017	-26.7	0.4	-27.3	0.4	0.6
2018	-24.5	0.6	-25.0	0.7	0.5
2019	-26.1	0.4	-26.6	0.4	0.5

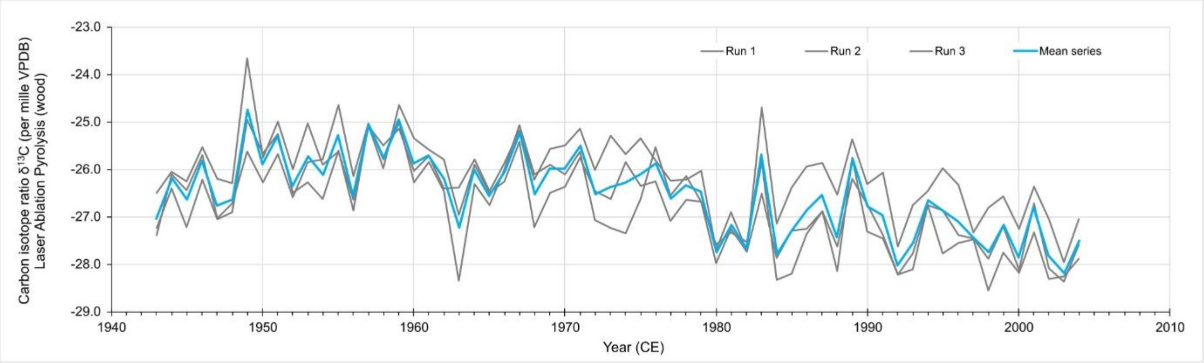

Figure S4. δ^{13}C values obtained from oak late wood sections by the new Laser Ablation Pyrolysis method. The three measurement series compare with a mean inter-series correlation of 0.80 and a mean standard deviation for the 62 triplicate measurements of 0.4 ‰ (min 0.0‰, max 1.1‰). Variation between the measurements may partly stem from matrix related issues, such as variation in wood composition across the latewood of this ring-porous species.

References

Saurer, M.; Sahlstedt, E.; Rinne-Garmston, K. T.; Lehmann, M. M.; Oettli, M.; Gessler, A.; Treydte, K. Progress in High-Resolution Isotope-Ratio Analysis of Tree Rings Using Laser Ablation. Tree Physiology 2023, 43 (5), 694–705. https://doi.org/10.1093/treephys/tpac141.
